# Self-Esteem and Academic Difficulties in Preadolescents and Adolescents Healed from Paediatric Leukaemia

**DOI:** 10.3390/cancers9060055

**Published:** 2017-05-24

**Authors:** Marta Tremolada, Livia Taverna, Sabrina Bonichini, Giuseppe Basso, Marta Pillon

**Affiliations:** 1 Department of Developmental and Social Psychology, University of Padua, Padua 35131, Italy; s.bonichini@unipd.it; 2 Faculty of Education, Free University of Bozen-Bolzano, Brixen-Bressanone 39042, Italy; livia.taverna@unibz.it; 3 Department of Child and Woman Health, Oncology Hematology Division, University-Hospital of Padua, Padua 35127, Italy; giuseppe.basso@unipd.it (G.B.); marta.pillon@unipd.it (M.P.)

**Keywords:** self-esteem, school achievement, preadolescents, adolescents, leukaemia, survivors

## Abstract

Adolescents with cancer may demonstrate problems in their self-esteem and schooling. This study aims to screen the preadolescents and adolescents more at risk in their self-esteem perception and schooling difficulties post-five years from the end of therapy. Twenty-five paediatric ex-patients healed from leukaemia were recruited at the Haematology-Oncologic Clinic (University of Padua). The mean age of the children was 13.64 years (Standard Deviation (SD)) = 3.08, range = 10–19 years), most were treated for acute lymphoblastic leukaemia (ALL) (84%) and relatively equally distributed by gender. They filled in the Multidimensional Self-Esteem Test, while parents completed a questionnaire on their child’s schooling. Global self-esteem was mostly below the 50 percentile (58.5%), especially regarding interpersonal relationships (75%). An independent sample *t*-test showed significant mean differences on the emotionality scale (*t* = 2.23; degree of freedom (df) = 24; *p* = 0.03) and in the bodily experience scale (*t* = 3.02; df = 24; *p* = 0.006) with survivors of Acute Myeloid Leukaemia (AML) having lower scores. An Analysis of Variance (ANOVA) showed significant mean differences in the bodily experience scale (*F* = 12.31; df = 2, *p* = 0.0001) depending on the survivors’ assigned risk band. The parent reports showed that 43.5% of children had difficulties at school. Childhood AML survivors with a high-risk treatment were more at risk in their self-esteem perceptions. Preventive interventions focusing on self-esteem and scholastic wellbeing are suggested in order to help their return to their normal schedules.

## 1. Introduction

Due to advances in chemotherapy and supportive care, about 82% of patients with childhood cancer have shown a dramatic increase in five-year survival [1]. There are long-term physiological and psychological sequelae of these treatments that may not manifest until paediatric survivors become adolescents [2,3], although other studies [4,5,6] suggest in these ex-patients a best psycho-social wellbeing than sane peers.

Literature on this topic is not consistent because quality of life is a complex construct, and studies dealing with this issue have used different instruments. In the paediatric cancer survivor research field, the major areas are: physical-functional wellbeing, social adaptation and psychological wellbeing. Healed patients seem to experience effects on their development (growth, cognitive and pubertal development) [7,8], on specific organs [9], or second neoplasms [10]. Socially, outcomes can involve school and job choices [11], social and love relationships [12] and family relations [13]. Psychologically, there are consequences such as: post-traumatic stress disorder [14], and effects on identity development [15] and body image [16].

One important area of psychological outcomes that has not yet been studied widely is the impact of the illness experience on self-esteem in preadolescents and adolescents healed from cancer. Self-esteem or self-concept could be defined as “a set of self-attitudes that reflects a description and an evaluation of one’s own behavior and attributes” [17]. It evolves in different environmental contexts in which children and adolescents are found to act, presenting a multidimensional nature that is captured through six scales that will be adopted in this study [18]: interpersonal relationships, environment control competence, emotionality, academic success, family life, and bodily experience. These dimensions show approximately the same importance level in building self-identity, even if the influential levels vary from person to person. The self-concepts of boys and girls are built from cognitive-behaviour patterns learned over time through interactions with specific environmental contexts. As these patterns act in various environments, the children assess the effectiveness of their actions. With increasing age, children’s evaluative behaviours become more and more coherent with each specific context and that consistency creates self-esteem related to the context. Self-concept is a construct that is probably influenced by a variety of factors such as cognition, neuropsychological factors, psychosocial factors, development, and emotion.

A chronic illness during childhood such as cancer, which affects health, physical appearance and opportunities for social interaction, could affect the self-concept of a person. Adolescents with cancer may face added burdens in the process of creating an identity and self-esteem [19]. Changes in self-esteem may involve psychological adjustment problems and difficulties in relationships or schooling, with negative self-concept being predictive of survivors with adjustment problems. The potential psychosocial problems for survivors, such as problems with schooling and employment, difficulties in forming relationships and adverse changes in appearance, can result in a possible lack of self-esteem [20]. A moderately increased risk of behavioural and educational problems was found in children with Acute Lymphoblastic Leukaemia (ALL). School performance was poorer in children with ALL attending primary school, compared with same-age peers; teacher-rated behaviour and mathematics performance was correlated with attention function in children with ALL. An excess of problematic behaviours and underperformance at school was found in the ALL high-risk (HR) group compared with the standard-risk group [21]. Negative outcomes in neuropsychological functioning were observed most frequently in survivors of acute lymphoblastic leukaemia and brain tumours [22]. The neurodevelopmental sequelae in childhood cancer survivors can have a range of effects on their psychological adjustment and quality of life as they reintegrate into school and social settings [23]. Areas that have been found to be problematic for paediatric survivors include academic achievement [24], employment difficulties [25], and impaired or decreased social relationships [26]. Children treated for leukaemia can have serious psychological and cognitive long-term sequelae due to cancer treatments that can influence their academic achievement [27]. There is a growing amount of literature on the presence of deficits in their executive functions such as attention, working memory, speed of processing, visuo-motor coordination or sequencing abilities, especially in children treated with a high-risk therapy for leukaemia caused by radiotherapy (RT), steroids and high dose of methotrexate (MTX) [28,29]. ALL survivors with neurocognitive deficits are at risk for poor Quality of Life (QOL), with broad implication for their physical, social, and school functioning [30]. Survivors of childhood acute lymphoblastic leukaemia are at risk for neurocognitive deficits that affect development in adolescence and young adulthood, and influence educational attainment and future independence. Adolescent survivors with cognitive or behaviour problems and those with learning problems are less likely to graduate from college as young adults than adolescent survivors without cognitive or behaviour problems [31].

Behavioural and social competences are important for a successful transition from cancer therapy to survivorship, and for a successful adult life. Survivors are reported to be withdrawn and socially isolated, and to have fewer same-age friends than their peers [32]. Seitzman et al. [33] reported a significantly lower global self-worth in ALL survivors than sibling controls, even when 81% of survivors had positive self-concepts. Significant differences in self-esteem were also found by Speechley et al. [34], where childhood cancer survivors reported lower levels compared to controls who had no history of cancer. Hill et al. [35] found that survivors who had received both Central Nervous System (CNS) irradiation (Cranial Radiotherapy (CRT)) and intrathecal methotrexate (IT-MTX) had significantly worse body image, lower school achievement, and worse overall adjustment than those who received IT-MTX without CRT. Some problematic areas in paediatric survivors related to psychological wellbeing included self-concept, self-esteem and identity [15]. A positive self-concept is a significant factor in overall good mental health and psychological wellbeing, and is considered by many psychologists as a basic psychological need [36,37,38].

Adolescence is often described as an important and sometimes vulnerable period in the development of body image (BI), an important issue that is also positively linked with the self-esteem construct [39]. BI assessed systematically among cancer survivors [40] suggested that factors such as peer/social support and age at diagnosis may affect BI, which should be conceptualised not only as an outcome variable, but also as a potentially important predictor of long-term psychological adjustment.

This study screened the preadolescents and adolescents most at risk in their self-esteem perception, and of experiencing schooling difficulties five years after the cessation of therapy, and identified the possible socio-demographic and disease risk factors. The cut-off of five years from cessation of therapy was chosen taking into consideration Italian Association for Pediatric Hematology-Oncology Acute Lymphoblastic Leukaemia protocol (AIEOP BFM ALL 2000) because, at this time point, the relapse risk or second tumour onset become minimal and the patients could be considered healed.

1. We expected to find lower global self-esteem in these boys and girls than the norms, as suggested by some studies [33,34], specifically in interpersonal and bodily experience areas, where the previous studies have found more difficulties [12,40].
2. We presumed that ex-patients with more aggressive therapy, including CRT (Acute Myeloid Leukaemia AML and ALL-HR), would report lower self-esteem scores, as suggested in the study of Hill et al. [35].
3. We expected that females would report more self-esteem problems, according to the findings of a study on adolescents during cancer treatments [41].
4. We hypothesized that we would find several schooling difficulties (especially in attention) and relationship problems [21,32].

## 2. Results

### 2.1. Self-Esteem Scoring in Preadolescents and Adolescents

Initially, we determined the standard scores of each of the six scales and the total scale, after which we calculated confidence intervals and percentile ranks. Finally, we created for each child a summary chart of self-esteem levels for each dimension. Table 1 shows the descriptive statistics of the self-esteem scales.

**Figure 1:**
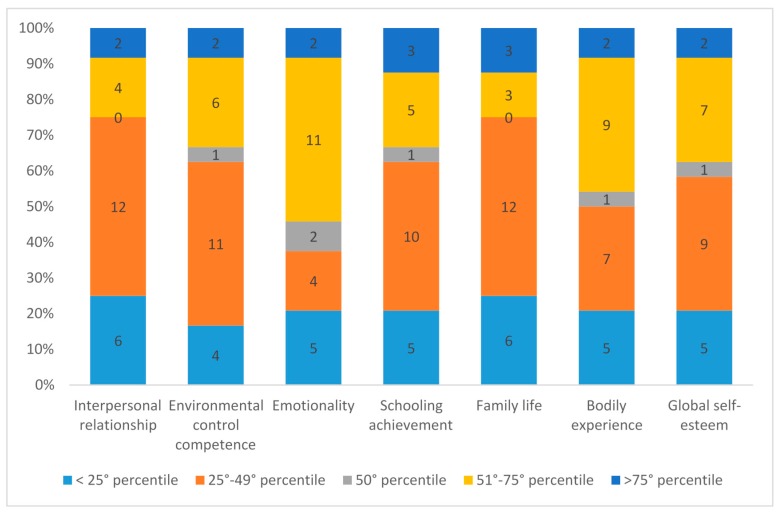
Self-esteem delays reported by preadolescents and adolescents.

Global self-esteem reported by boys and girls was mostly below the 50 percentile (58.5%), especially on the following scales: interpersonal relationships (75%), environmental control competence (62.5%), academic success (62.5%), family life (75%), bodily experience (50%) and emotionality (37.5%). Figure 1 shows these results.

Interpersonal relationships and family life were the lowest reported scales of self-esteem, followed by environmental control competence and academic success, where two-thirds of the ex-patients reported levels of self-esteem below the norms. Table 2 shows the significant correlations between self-esteem scales. Interpersonal relationship self-esteem was closely related to the emotionality, family life and bodily experience scales. Environmental control competence was associated with emotionality, bodily experience and academic success. Bodily experience was associated with all the scales except family life.

### 2.2. Schooling Situation and Difficulties Reported by Parents

Table 3 shows the schooling situation reported by parents. Parents reported the most frequent difficulties met by their children as: feeling uncomfortable with teachers; problems earning strategies to study; concentration problems in the disciplines that they don’t like; anxiety if they don’t feel ready; difficulty maintaining attention at school and during homework; isolation from peers and difficulty concentrating in the first two years after the end of the treatments; difficulties in logic-mathematics, difficulties in learning multiplication tables by memory, and problems making associations throughout the several disciplines and in underestimating the required commitment.

Parental reports on their children’s school functioning revealed that 43.5% had difficulties at school, for example in concentration or socialization. Although most also had lessons in hospital or at home (respectively 62.5%, and 66.7%), the school programs were not always the same as those of their school peers (21.4%), or were not agreed with the residential school (50%).

### 2.3. Associations between Self-Esteem Scoring and Socio-Demographic Variables

We wanted to verify, using Spearman’s correlations, whether some socio-demographic variables were related to the self-esteem scales of Multidimensional Self-Esteem Test (TMA). Gender and current age weren’t significantly associated with any of the six self-esteem scales: interpersonal relationships (*r* = −0.15; *p* = 0.47; *r* = −0.25; *p* = 0.21), environmental control competence (*r* = 0.13; *p* = 0.52; *r* = −0.85; *p* = 0.68), school achievement (*r* = 0.011; *p* = 0.95; *r* = −0.15; *p* = 0.45), family life (*r* = −0.017; *p* = 0.93; *r* = −0.16; *p* = 0.41), bodily experience (*r* = 0.07; *p* = 0.42; *r* = −0.016; *p* = 0.94) and emotionality (*r* = 0.011; *p* = 0.95; *r* = −0.15; *p* = 0.45). We also considered whether the age at diagnosis affected self-esteem scores, but we did not obtain any significant results.

### 2.4. Associations between Self-Esteem Scoring and Illness Variables

**Figure 2:**
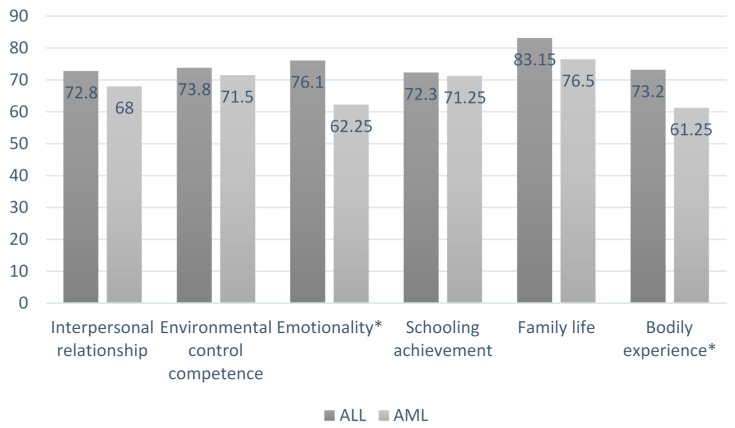
Significant mean differences of self-esteem scores along diagnosis type: Acute Lymphoblastic Leukaemia (ALL) vs. Acute Myeloid Leukaemia (AML).

The independent sample *t*-test showed significant mean differences in the emotionality scale (*t* = 2.23; degree of freedom (df) = 24; *p* = 0.03) and in the bodily experience scale (*t* = 3.02; df = 24; *p* = 0.006) depending on the survivor’s type of leukaemia. Examining the expected marginal means, survivors of AML showed lower scores in the emotionality (Mean (M) = 65.25; Standard Deviation (SD) = 7.84) and in the bodily experience (M = 61.25; SD = 6.47) self-esteem scales than ALL survivors (respectively, M = 76.10; SD = 8.76; M = 73.2; SD = 10.78) as shown in Figure 2. 

**Figure 3:**
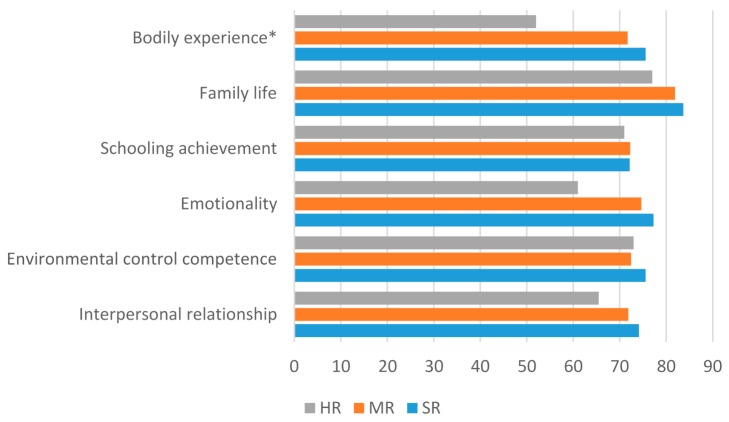
Significant mean differences of self-esteem scores along risk band: High Risk (HR), Medium Risk (MR), Standard-Risk (SR).

The Analysis of Variance (ANOVA) showed significant mean differences in the bodily experience scale (*F* = 12.31; df = 2, *p* = 0.0001) depending on the survivors’ assigned risk band (Standard Risk: SR; Medium Risk: MR; High Risk: HR). Bonferroni post hoc analysis identified the scores reported by children treated for a high risk leukaemia as significantly lower from those treated respectively for a standard-risk leukaemia (Mean difference = −23.57; SD = 4.78; *p* = 0.0001) and medium-risk leukaemia (Mean difference = −19.73; SD = 4.48; *p* = 0.001) (Figure 3).

## 3. Discussion

Self-esteem is a key construct in paediatric health psychology, closely associated with psycho-social wellbeing. It reflects a person’s overall subjective emotional evaluation of their own worth. It is a judgment of one’s self, as well as an attitude toward the self.

Self-esteem encompasses beliefs about one’s self, (for example, “I am competent”, “I am worthy”), as well as emotional states (i.e., triumph, despair, pride, shame). Self-esteem is attractive as a social psychological construct because researchers have conceptualised it as an influential predictor of outcomes such as academic achievement, happiness, and satisfaction in relationships. Self-esteem can apply specifically to a particular dimension (for example, “I believe I am a good student and feel happy about that”), or more globally (for example, “I believe I am a bad person, and feel bad about myself in general”). Maslow’s hierarchy of needs [42], which depicts self-esteem as one of the basic human motivations, suggested that people need both esteem from other people and inner self-respect. Both of these needs must be fulfilled in order for an individual to grow as a person and achieve self-actualisation.

The need for positive self-esteem plays an important role in preadolescents and adolescents who have been healed from leukaemia. The experience of a chronic disease such as cancer during childhood has an impact on the development and on psychological wellbeing of these paediatric patients. Some problems can emerge when these ex-patients return to their normal schedules [43], so it is interesting to investigate self-esteem and schooling difficulties when they are declared healed (five years after the end of therapy), when they are in the delicate developmental stage of pre-adolescence and adolescence.

Studies have reported more difficulties in several aspects of life: social relationships [26,40], body experience/image [12], academic achievement [24,28,44], and employment [25]. All of these areas are closely associated with the self-esteem construct, and with Bracken et al.’s [18] hierarchical model. The literature also noted that paediatric cancer survivors showed lower global self-esteem when comparing them with norms or with siblings [33,34].

In this study, we found clinically relevant low levels of self-esteem reported by preadolescents and adolescents when they were healed. Self-esteem was worse in relation to interpersonal relationships and family life, environmental control, competence and academic success. Bodily experience was also reported as low, and emotionality frequently did not show clinically relevant values. This result is also connected with the schooling difficulties reported by parents, which are related to social relationships with companions or with teachers, and to attention and concentration. Even when healed, these ex-patients reported a certain number of absences because their health conditions weren’t optimal for attending school. Reintegration into an educational context after a long period of therapy (generally more than one year) also requires a re-adaption process regarding instructional constraints, and maintaining concentration could be difficult for these adolescents.

Is it possible to identify ex-patients as more at risk of negative self-esteem perceptions? Previous studies have emphasised more aggressive therapy (i.e., including CRT) as a risk factor [30], so we hypothesised that ex-patients treated for a diagnosis of AML or ALL-HR, who underwent hematopoietic stem cell transplantation and total body irradiation, would report lower self-esteem levels. This hypothesis was confirmed, especially in some aspects of self-esteem: global scores, emotionality and bodily experience. Body image, emotionality and global self-esteem are negatively affected by an increase in the toxicity of therapy.

Our hypothesis that females might report lower self-esteem levels was not confirmed here, nor did we find differences when considering current age or age at diagnosis. Probably, the participant number is too limited, or the cancer experience could have flattened the normal differences between males and females. Socio-demographic factors seem to be less relevant in impacting self-esteem in cancer childhood survivors. Future studies with larger samples could help us to better understand this phenomenon.

The number of ex-patients who participated in this study was unfortunately limited, and more centres need to be involved to allow generalisability of results. However, the substantial heterogeneity in diagnosis and treatment that is evident within the patient group could be considered a strength of this study. Generally, the precedent studies put together several types of diagnosis [32], but the different treatment protocols (i.e., the duration of the cycles, intensity of chemotherapy, radiotherapy presence/absence, System Nervous Center prophylaxis presence/absence) could inevitably influence self-esteem and the schooling situation. Thus, in this study, the variability associated with the type of cancer is very controlled, so that other, more specific, medical and socio-demographic variables could be exclusively considered as possible associate factors of children’s self-esteem scales.

Another recommendation for future research is to include information from teachers about cancer paediatric patients that re-enter school [39], such as about their behaviour in the classroom, whether they demonstrate attentional and concentration problems, and the types of relationships they have with companions [40]. Preventive interventions focused on increasing self-esteem, school wellbeing and social adaptation should be improved to help paediatric cancer ex-patients go back to their normal schedules. The supportive intervention could be focused on two directions before the child’s school re-entry. The first intervention could be directed to the children though a psychotherapist, both individually or in group, on their feelings and emotions towards going back to school and to their normal schedules. The second intervention focus could be directed to the children environment: a supportive intervention on parenting could help to sustain the child during his integration program, and a preventive residential training work with his teachers and peers could give some practical indications to favour the child’s re-entry.

## 4. Materials and Methods

### 4.1. Participants

One hundred and twenty-two families recruited at the Haematology-Oncologic Clinic of the Department of Child and Woman Health, University of Padua, agreed to participate in the research-clinical project entitled “Family factors predictive short and long term adaptation and quality of life in children with leukaemia: A longitudinal study”. Families were contacted by a clinical psychologist during the first hospitalisation of their children, about one week after diagnosis. The project’s aims were explained and informed consent was requested. An in-depth interview and a battery of questionnaires were administered at several established time points during the therapy cycles.

The following inclusion criteria were used: children aged from 9 years to 19 years old because we wanted to assess self-esteem in preadolescents and adolescents and the questionnaire adopted here is validated for this age range, with a diagnosis of ALL or AML; time of administration (5 years from the end of therapy, when children are considered survivors for BFM ALL AIEOP 2000 protocol); no history of neurodevelopmental disorders such as learning or sensory disabilities with a basic level of reading comprehension (according to information derived from the schooling questionnaire given to parents) or other genetic syndromes. Forty patients matched these parameters. Eighty-two children did not participate for different reasons: 13 of them were not reached in their yearly follow-ups, 10 of them were not in the age range included in this project, 54 had not yet reached the amount of 5 years off of therapy, and 5 of them changed the hospital center.

From this sample, we assessed 25 preadolescents and adolescents healed from leukaemia (response rate: 62.5%) during their follow-up, five years from the end of their therapy. The rate of attrition (37.5%) was caused by several reasons: not giving back or incomplete questionnaires (33%), not contacted at the follow-up for logistic assessment problems (i.e., psychologist not present for other contemporary appointments, unexpected change of follow-up date, patients not coming at the medical follow-up; 57%), dropped out from the study because they did not want to think to the illness anymore (10%). Their mean age was 13.64 years (SD = 3.08, range = 10–19 years), most were treated at a mean age of 6.95 (SD = 3.52) for ALL (84%) and 12% were treated for AML, 11 were females and 14 were males. Dealing with the therapy intensity, 8 children followed a standard-risk protocol, 15 a medium risk, and 2 a high risk one. Only 4 of them had a relapse and 3 underwent Hematopoietic Stem Cell Transplantation (HSCT).

All parents were Caucasian, the mother’s mean age was 44.50 years (SD = 5.13) and averaged 13 years of schooling (52.2%). The father’s mean age was 48.70 (SD = 4.13), and they mostly had achieved two education levels: either 8 (45.5%) or 13 years of schooling (45.5%). Parent incomes were mostly average (50%), equally distributed between high (33.3%) and low (16.7%) incomes for Italian norms, but above poverty. The ex-patients had at least one sibling (61.9%), or two or more siblings (23.8%) and only 14.3% were only children.

### 4.2. Procedure

Ethics approval was obtained from “Hospital of Padua Ethical Committee”. The patients and their families were contacted by a clinical psychologist during the first hospitalisation of the children, about one week after diagnosis. The project aims were explained and informed consent was requested. Informal contacts with the participants were kept up on a daily basis, to provide support and motivation for the project. The participants were informed that they were free to drop out at any time of the study. The preadolescents and adolescents with leukaemia who had completed therapy five years previously were contacted again and the Multidimensional Self-Esteem Test and a schooling questionnaire were administered at the Day Hospital (DH) of the clinic. Before the assessments, the psychologist contacted the parents by telephone to agree about meetings. Medical and socio-demographic information was also collected.

### 4.3. Instruments

#### 4.3.1. Multidimensional Self-Esteem Test

This questionnaire was built according to the self and self-esteem definitions of Shavelson et al. [45]: internal constitution, multidimensional nature, hierarchical structure, stability over time, relations with the psychological development of the person, assessing mode, and significant difference compared to other psychological constructs. This test uses six topics regarding which a child makes a self-estimation: the interpersonal area, environmental control area, emotive area, schooling area, family area, and body area. These areas are correlated, contributing to the general vision of self.

The Multidimensional TMA theoretical model assumes that self-esteem is developed in a structured hierarchical manner according to the principles of learning skills. A learned response style that reflects the assessment made by the individual of their experience and past behaviour could also predict their future behaviour. Self-esteem is assessed by asking the child/adolescent about their degree of agreement with a series of statements that describe their influence on their environment in different areas. This psychological construct contains all the learned assessments that individuals make about themselves based on their successes and failures, personal reinforcement experiences and the ways in which others have reacted to, and interacted with them.

The TMA has been developed as a comprehensive assessment tool for children/adolescents aged between 9 years and 19 years. It can be used to evaluate both global self-esteem and its specific six dimensions. The TMA has been validated for the Italian population [18] adopting an accepted standardised scoring to facilitate the interpretation and integration of scores with other psycho-educative tests. Psychometric characteristics of TMA are good, showing a Cronbach’s alpha > 0.96, standard error of 2.12 and a test–retest’s *r* > 0.90. In addition, content and concurrent validity are appropriate comparing the TMA scores with other self-esteem surveys (Cooper Smith Self Esteem Inventory and Piers-Harris Self-Concept Scale) and with a control group. This test was standardized on a sample of 2501 children aged 9–19 years and the manual reports the normative values.

#### 4.3.2. Socio Economic Status Questionnaire

Parental education and occupational status were measured. Data was collected on education (the number of years of school achievement), type and average hours of job, and economic status.

#### 4.3.3. Schooling Situation

School absences, school involvement at the hospital and at home during illness isolation, and school marks were reported by parents because they could be considered the most reliable witnesses about the period of their children hospitalization, while ex-patients could not remember this type of information. We verified if the children could fill in the self-report questionnaire by themselves, with a sufficient level of reading and comprehension.

### 4.4. Statistical Analysis

We ran descriptive statistics to show the child’s self-esteem scores five years after stopping therapy, and we determined the standard scores of each of the six scales and the total scale. Then, we determined confidence intervals and percentile ranks.

The data were checked for normality adopting the Kolmagorov–Smirnov and Shapiro–Wilk tests. Data distribution was normal, so we decided to use parametric statistics.

Preliminary Pearson’s correlations were run to identify the possible significant associations between the variables. A series of independent sample *t*-tests and ANOVAs were run to identify medical variables significantly associated with the self-esteem scales. These variables included in the analysis were: type of diagnosis (ALL, AML), HSCT (presence vs. absence) and treatment risk band (SR, MR, HR). We adopted the Bonferroni post hoc adjustment for multiple comparisons and we controlled that the variances obtained were homogeneous. All data were analysed using SPSS version 22 (SPSS Inc., Chicago, IL, USA).

## 5. Conclusions

The main findings of this study were the alarming low levels of self-esteem reported by preadolescents and adolescents when they were considered healed. Self-esteem was worse in relation to interpersonal relationships and family life, environmental control, competence and academic success and bodily experience, while emotionality frequently did not show clinically relevant values. This result is also related with the schooling difficulties reported by parents, such as the problematic social relationships with companions or with teachers, and the attention and concentration keeping problems. The identified predictive factors for a more negative self-esteem perceptions were the diagnosis of AML and the high intensity of therapy. Recommendations for future research and clinical suggestions were given.

## Figures and Tables

**Table 1:** Descriptive statistics of the self-esteem scales.

Percentile Ranks	*N*	M	SD	Minimum	Maximum
*Interpersonal relationship*	25	38.83	20.28	5	83
*Environmental control competence*	25	44.25	22.77	5	88
*Emotionality*	25	50.37	24.84	8	93
*Schooling achievement*	25	43.96	23.70	11	94
*Family life*	25	40.92	22.58	8	96
*Bodily experience*	25	45.17	22.83	4	87
*Global self-esteem*	25	42.71	22.09	11	91

SD: Standard Deviation. M: Mean.

**Table 2:** Spearman’s correlation between the self-esteem scales.

	*Interpersonal Relationship*	*Environmental Control Competence*	*Emotionality*	*Schooling Achievement*	*Family Life*
*Environmental control competence*	*r* = 0.33				
	*p* = 0.10				
*Emotionality*	*r* = 0.622 **	*r* = 0.66 **			
	*p* = 0.001	*p* = 0.001			
*Schooling achievement*	*r* = 0.28	*r* = 0.41 *	*r* = 0.41 *		
	*p* = 0.16	*p* = 0.04	*p* = 0.04		
*Family life*	*r* = 0.50 *	*r* = 0.12	*r* = 0.51 **	*r* = 0.37	
	*p* = 0.01	*p* = 0.55	*p* = 0.009	*p* = 0.07	
*Bodily experience*	*r* = 0.50 *	*r* = 0.53**	*r* = 0.62 **	*r* = 0.53 **	*r* = 0.39
	*p* = 0.01	*p* = 0.006	*p* = 0.001	*p* = 0.006	*p* = 0.053

* *p* ≤ 0.05 (two-tailed), ** *p* ≤ 0.01 (two-tailed).

**Table 3:** School situation reported by parents.

	Yes				No
**During his school career did he/she encounter any difficulties?**	*n* = 11				*n* =13
**During his school career did he/she repeat year/s?**	*n* = 1				*n* = 23
**During therapies did he/she participate in school activities at hospital?**	*n* = 15				*n* = 9
	Program agreed with the teachers of the school and equal to their peers: 28.6%	Program different from their peers: 21.4%		Program not agreed with the teachers of the school: 50%	
**During therapies did he/she had participated in school activities at home?**	*n* = 16				*n* = 8
	Program agreed with the teachers of the school and equal to their peers: 75%		Program different from their peers: 25%		
**How long has the child started to go to school after the diagnosis communication?**	6 months: *n* = 5				
	12 months: *n* = 10				
	18 months: *n* = 5				
	24 months: *n* = 2				
	more than 24 months: *n* = 2				
**Number of absences in the last scholastic year**	No absence: *n* = 1				
	Only for important visits: *n* = 1				
	4–10 h: *n* = 3				
	2–4 days: *n* = 6				
	5–7 days: *n* = 4				
	9–10 days: *n* = 4				
	20 days: *n* = 1				
	60 days: *n* = 1				
	No response: *n* = 3				
